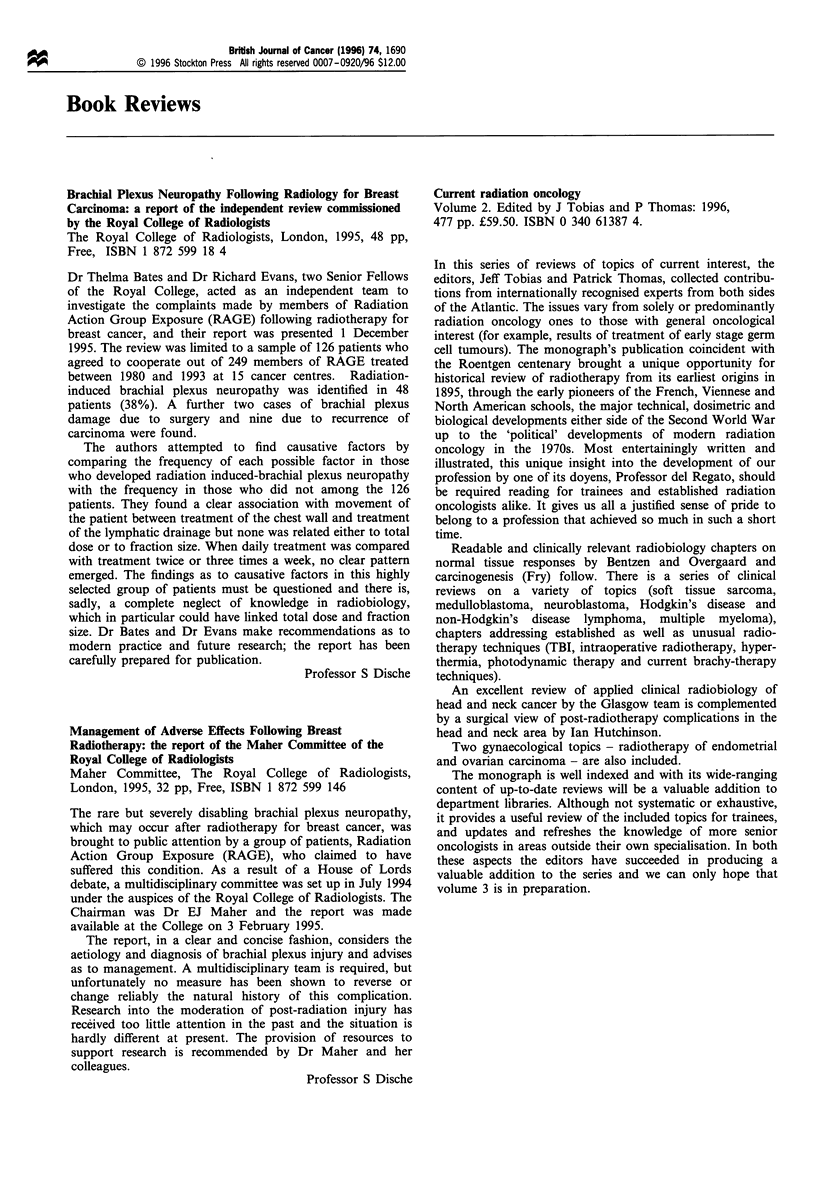# Management of Adverse Effects Following Breast Radiotherapy: the report of the Maher Committee of the Royal College of Radiologists

**Published:** 1996-11

**Authors:** S Dische


Professor S Dische

**Management of Adverse Effects Following Breast Radiotherapy: the report of the Maher Committee of the Royal College of Radiologists**
Maher Committee, The Royal College of Radiologists, London, 1995, 32 pp, Free, ISBN 1 872 599 146

The rare but severely disabling brachial plexus neuropathy, which may occur after radiotherapy for breast cancer, was brought to public attention by a group of patients, Radiation Action Group Exposure (RAGE), who claimed to have suffered this condition. As a result of a House of Lords debate, a multidisciplinary committee was set up in July 1994 under the auspices of the Royal College of Radiologists. The Chairman was Dr EJ Maher and the report was made available at the College on 3 February 1995.

The report, in a clear and concise fashion, considers the aetiology and diagnosis of brachial plexus injury and advises as to management. A multidisciplinary team is required, but unfortunately no measure has been shown to reverse or change reliably the natural history of this complication. Research into the moderation of post-radiation injury has received too little attention in the past and the situation is hardly different at present. The provision of resources to support research is recommended by Dr Maher and her colleagues.